# Exploratory Study to Identify Radiomics Classifiers for Lung Cancer Histology

**DOI:** 10.3389/fonc.2016.00071

**Published:** 2016-03-30

**Authors:** Weimiao Wu, Chintan Parmar, Patrick Grossmann, John Quackenbush, Philippe Lambin, Johan Bussink, Raymond Mak, Hugo J. W. L. Aerts

**Affiliations:** ^1^Department of Radiation Oncology, Dana-Farber Cancer Institute, Brigham and Women’s Hospital, Harvard Medical School, Boston, MA, USA; ^2^Department of Biostatistics, Harvard T.H. Chan School of Public Health, Boston, MA, USA; ^3^Department of Radiology, Dana-Farber Cancer Institute, Brigham and Women’s Hospital, Harvard Medical School, Boston, MA, USA; ^4^Research Institute GROW, Maastricht University, Maastricht, Netherlands; ^5^Department of Biostatistics and Computational Biology, Dana-Farber Cancer Institute, Boston, MA, USA; ^6^Department of Radiation Oncology, Radboud University Medical Center, Nijmegen, Netherlands

**Keywords:** quantitative imaging, radiomics, lung cancer histology, computational science, feature selection

## Abstract

**Background:**

Radiomics can quantify tumor phenotypic characteristics non-invasively by applying feature algorithms to medical imaging data. In this study of lung cancer patients, we investigated the association between radiomic features and the tumor histologic subtypes (adenocarcinoma and squamous cell carcinoma). Furthermore, in order to predict histologic subtypes, we employed machine-learning methods and independently evaluated their prediction performance.

**Methods:**

Two independent radiomic cohorts with a combined size of 350 patients were included in our analysis. A total of 440 radiomic features were extracted from the segmented tumor volumes of pretreatment CT images. These radiomic features quantify tumor phenotypic characteristics on medical images using tumor shape and size, intensity statistics, and texture. Univariate analysis was performed to assess each feature’s association with the histological subtypes. In our multivariate analysis, we investigated 24 feature selection methods and 3 classification methods for histology prediction. Multivariate models were trained on the training cohort and their performance was evaluated on the independent validation cohort using the area under ROC curve (AUC). Histology was determined from surgical specimen.

**Results:**

In our univariate analysis, we observed that fifty-three radiomic features were significantly associated with tumor histology. In multivariate analysis, feature selection methods ReliefF and its variants showed higher prediction accuracy as compared to other methods. We found that Naive Baye’s classifier outperforms other classifiers and achieved the highest AUC (0.72; *p*-value = 2.3 × 10^−7^) with five features: Stats_min, Wavelet_HLL_rlgl_lowGrayLevelRunEmphasis, Wavelet_HHL_stats_median, Wavelet_HLL_stats_skewness, and Wavelet_HLH_glcm_clusShade.

**Conclusion:**

Histological subtypes can influence the choice of a treatment/therapy for lung cancer patients. We observed that radiomic features show significant association with the lung tumor histology. Moreover, radiomics-based multivariate classifiers were independently validated for the prediction of histological subtypes. Despite achieving lower than optimal prediction accuracy (AUC 0.72), our analysis highlights the impressive potential of non-invasive and cost-effective radiomics for precision medicine. Further research in this direction could lead us to optimal performance and therefore to clinical applicability, which could enhance the efficiency and efficacy of cancer care.

## Introduction

Lung cancer is the leading cause of cancer-related deaths worldwide with 150,000 deaths per year in US (1). Lung cancer is clinically divided into two groups: small cell lung cancer (SCLC, ~25%) and non-small cell lung cancer (NSCLC, ~75%) (2). The most common histological subtypes of NSCLC are adenocarcinoma (~38%) and squamous cell carcinoma (~20%) (3). These two subtypes have distinct histologic features, i.e., squamous cell carcinoma is associated with intercellular bridging and individual cell keratinization, whereas glandular architecture is prominent for adenocarcinoma (3, 4). Histological classification of lung cancer provides important information about tissue characteristics and anatomical location. Adenocarcinoma often develops at the periphery of the lungs, while squamous carcinoma is generally located more centrally (5). Differences have also been found in expression of glycolysis and hypoxia-related markers between histological subtypes, which suggests histology-specific glucose metabolism in NSCLC (5, 6). In addition, histological tumor classification could determine the optimal treatment and/or therapy strategies (7). Recent advancement in the therapy for lung cancer is characterized by discovery of targetable mutations and histology-based therapeutic regimen selection (7, 8). For example, Pemetrexed is the preferred treatment for stage IV lung adenocarcinoma, whereas bevacizumab is not recommended for squamous carcinoma due to the risk of pulmonary hemorrhage observed in phase II trials (8–10). It has also been shown that treatment for stage III lung cancer patients with squamous carcinoma has significant improvement in survival with cisplatin/gemcitabine versus cisplatin/pemetrexed, but for adenocarcinoma patients, the latter treatment provides superior survival rate (11, 12). More importantly, histology classification increases the likelihood of identifying patients with targetable mutations like EGFR mutations, which occurs primarily in adenocarcinoma (4).

In routine clinical practice, the most common way of classifying tumor histology is through the histopathological analysis of tumor tissues via biopsy. This is clinically limited by the inherent risk of invasive procedures as well as poor time and cost efficiency (8). Therefore, automatic, non-invasive, and cost-effective alternatives are desired. Medical imaging provides promising opportunities in this regard. It assesses the tumor tissue characteristics non-invasively. Furthermore, it is relatively cost-effective and is already a routinely used clinical practice for oncologic diagnosis, staging, and treatment guidance (13–15).

Radiomics, a high throughput data mining approach, can exploit the non-invasive medical image data (14). It focuses on extracting a large number of quantitative imaging features, which can provide a detailed and comprehensive characterization of the tumor phenotype (8). Several studies have shown the prognostic/predictive power of radiomic features in different cancer types by using different medical imaging modalities like CT (16, 17), MRI (18), PET (15, 19, 20), and US (21). It has been shown that radiomic features are associated with several clinically relevant factors, such as tumor stage (22), tumor metabolism (23), overall patient survival (17, 24), metastasis (13), treatment response (25), and the underlying gene expression profiles (26, 27). These associations can be leveraged to build efficient and effective prediction/prognostic models. Therefore, radiomics is a promising field providing a non-invasive and cost-effective way for personalized medicine.

A limited number of studies have investigated the association of radiomic features and NSCLC tumor histology (22, 28). Most of them used a clustering-based unsupervised approach for associating radiomic features with tumor histological subtypes. However, in order to achieve higher prediction accuracies, supervised methods are generally preferred over unsupervised approaches if labeled data is available. Furthermore, like any other high-throughput data mining approach, radiomics also falls prey to the curse of dimensionality, which means we would need to get an enormous amount samples due to high dimensional radiomic features (29). Feature/variable selection is one of the solutions to this problem (30). Many feature selection methods have been proposed in machine learning literature, which should be applied for radiomics-based predictive analyses (31).

In this study, we investigated 24 commonly used feature selection methods and 3 supervised machine-learning classification methods for the prediction of lung cancer histologic subtypes, using independent training and validation cohorts from two different institutions. We first reduced the radiomic feature space into a non-redundant subspace by using correlation-based feature elimination. Second, we applied 24 (Information Gain, Gain Ratio Gini Index, MDL, DKM, ReliefF, and their variants) univariate filter-based feature selection methods to the resultant non-redundant feature subset. We chose these filter-based methods because of their popularity in the literature and their high computational efficiency.

The main objective of this study was to investigate the association between radiomic features and lung tumor histology. We employed machine-learning methods to build radiomics-based multivariate classifiers for the prediction of tumor histology. Non-invasive and cost-effective radiomic data could improve the histological classification and hence the treatment/therapy, which in general could have a large impact in cancer care. With improving image feature extraction techniques, a higher accuracy in classification is expected to achieve. This work will serve as a promising prognostic tool for informing treatment choice and fostering personalized therapy for lung cancer patients.

## Materials and Methods

### Datasets

We used two NSCLC cohorts collected at different institutions in the Netherlands. The training dataset (Lung1) contains 198 patients (mean age 69.5, range 34–88 years) with pathologically confirmed adenocarcinoma (*n* = 152) or squamous carcinoma (*n* = 51), UICC stages I–IIIb, treated with radical radiotherapy or with chemoradiation at MAASTRO Clinic in Maastricht in the Netherlands. Classification of the tumors as either adenocarcinoma or squamous cell carcinoma was based on hemotoxylin and eosin (H&E) staining according to the World Health Organization (WHO) classification of malignant lung tumors. Experienced radiation oncologists using a standard clinical delineation protocol performed delineation based on fused PET-CT imaging.

The test dataset (Lung2) has 152 patients (mean age 65.6, range 41–86 years) with pathologically confirmed adenocarcinoma (*n* = 62) or squamous carcinoma (*n* = 90), stages (I–IVa), treated at Radboud University Medical Center (RUMC) in Nijmegen, the Netherlands, between February 2004 and October 2011. Histological classification was based on H&E staining according to the WHO classification of malignant lung tumors. Manual delineations of the CT-scans were available for all included patients in the two datasets. Further details regarding the two data sets can be found in a previous paper (32). The Institutional Review Board of the Maastricht University Medical Center (MUMC+) and the Institutional Review Board of the RUMC waved review due to the retrospective nature of this study (32).

### Radiomic Features

Tumor phenotypic characteristics were quantified by extracting 440 3D radiomic features from the segmented tumor regions of pretreatment CT images (32). All radiomic features can be divided into three groups: (1) Intensity: these features quantify the density of the tumor region on the CT image from the first-order histogram of voxel intensities. (2) Shape: these features quantify the 3D geometric properties of the tumors. (3) Texture: textural features quantify the intratumor heterogeneity by using the gray level cooccurrence (GLCM) and gray level run length matrices (GLRLM). Intensity and textural features were also computed after applying 3D wavelet transformations (coiflet filters) to the original image. Matlab R2012b was used for the image analysis, and radiomic features were automatically extracted using Matlab R2012b. Details about the image analysis as well as the mathematical definition of the radiomic features can be found in previous literature (32). All radiomic data were centered and scaled before performing the analysis.

### Univariate Analysis

The association between the radiomic features and histological subtypes was assessed using the area under the receiver operating characteristic curve (ROC curve) (33). We computed AUC for all the features in a univariate manner. Significance was estimated using a random permutation test with iteration of 1000. The analysis was performed using R package survcomp (34).

### Feature Selection

Like any other high throughput approach, radiomics also has highly redundant feature space. So, if we just rank features based on feature relevance, it is likely that highly correlated features have similar rank and they end up together in the selected feature subset (35). Several studies have discussed this issue with respect to filter-based feature selection methods (36, 37). Besides, correlated features can cause the classifiers to be sensitive to small changes in the datasets. This could cause a severe problem in cohorts with a different structure of collinearity (38).

To address this problem, we used a simple two-stage feature selection method by combing correlation-based feature elimination and univariate feature selection. In the first stage, we eliminated highly correlated features using a correlation matrix. We calculated column-wise average absolute correlation $C=\frac{1}{n}\sum_{j}\;c_{\mathrm{ij}}$ for each feature. A threshold M is set for elimination, that is, for each pair-wise correlation *c_ij_* that exceed M, we removed the feature with higher column-wise average absolute correlation *C*. By eliminating those highly correlated features, we are left with “non-redundant” set of features. This process was implemented by R package “corrplot” with “findCorrelation” function (39).

In the second stage, we applied univariate feature selection methods to the non-redundant feature set generated in the first stage and chose top-ranked features. Feature ranking methods that we have used in this study are Gini index, Information Gain, Gain ratio, MDL, DKM and RelifF, and their variants (40, 41). Detailed description of these methods can be found in documentation of R package “CORElearn” (42).

Most of the used feature selection methods rank features based on their discriminating abilities between classes. Assume we have a set of *m* dimensional feature vectors *A* = (*A*_1_, *A*_2_, …, *A_m_*) and *c* number of labeled classes τ = (τ_1_, τ_2_, …, τ_*c*_). They evaluate each feature by the purity gained by adding the split on *A_i_* = *a_i,j_* Split is the partitioning of samples according the values of feature at evaluation. *p*(τ*_i_*) is the probability of class τ*_i_*, and $p\left(\tau_{i}|a_{i,j}\right)$ is the probability of class τ*_i_* conditioned on the feature *A_i_* has value *a_i,j_* (43). These information theory-based feature selection approaches and their corresponding scoring functions are described in Table 1. The derivatives and equations are cited and summarized from the Ref. (40, 43).

**Table 1:** **Feature filtering methods and corresponding scoring schemes**.

Feature filteringmethods	Scoring function
Information gain (44)	$\text{IN }\left(A_{i}\right)=\sum_{i=1}^{c}\;p\left(\tau_{i}\right)\;\log\;p\left(\tau_{i}\right)-\underset{j=1}{\overset{a_{\mathrm{mi}}}{\sum }}\;\underset{i=1}{\overset{c}{\sum }}\;p\left(\tau_{i}|a_{i,j}\right)\text{ log }p\left(\tau_{i}|a_{i,j}\right)$
Gain ratio (45)	$\text{GR }\left(A_{i}\right)=\frac{\sum_{i=1}^{c}p\left(\tau_{i}\right)\log p\left(\tau_{i}\right)-\sum_{j=1}^{a_{\mathrm{mi}}}\sum_{i=1}^{c}p\left(\tau_{i}|a_{i,j}\right)\text{log}p\left(\tau_{i}|a_{i,j}\right)}{\sum_{j=1}^{a_{\mathrm{mi}}}p\left(a_{\mathrm{ij}}\right)\log p\left(a_{\mathrm{ij}}\right)}$
Gini index (46)	$\text{GI }\left(A_{i}\right)=\sum_{i=1}^{c}\;p^{2}\left(\tau_{i}\right)-\sum_{j=1}^{a_{mi}}\sum_{i=1}^{c}\;p^{2}\left(\tau_{i}|a_{i,j}\right)$
MDL (47)	$\begin{array}{cc} \text{MDL }\left(A_{i}\right)\; & =\frac{1}{n}\log_{2}\left(\begin{array}{c} n\\ n_{1.},\ldots ,n_{c}. \end{array}\right)\\ \; & \;-\sum_{j=1}^{a_{mi}}\;\left(\begin{array}{c} n_{.j}\\ n_{1j},\ldots ,n_{\mathrm{cj}} \end{array}\right)+\log_{2}\left(\begin{array}{c} n+c+1\\ c-1 \end{array}\right)\\ \; & \;-\sum_{j=1}^{a_{mi}}\;\left(\begin{array}{c} n_{.j}+c-1\\ c-1 \end{array}\right) \end{array}$
	where *n_ij_* is number of samples from class ith with jth value of features
DKM (40)	$\begin{array}{cc} \text{DKM}\left(A_{i}\right) & =\sum_{i=1}^{c}2\sqrt{p\left(\tau_{\text{max}}\right)(1-p\left(\tau_{\text{max}}\right)}\\ & \;-\sum_{j=1}^{a_{mi}}\sum_{i=1}^{c}\;\sqrt{p\left(\tau_{\text{max}}|a_{\mathrm{ij}}\right)(1-p\left(\tau_{\text{max}}|a_{\mathrm{ij}}\right)} \end{array}$
	where $p\left(\tau_{\text{max}}\right){=}_{i=1\;to\;c}^{\max}\;p\left(\tau_{i}\right)$ represents most probable class value

#### ReliefF

ReliefF (44) evaluates partitioning power of features based on how well their values distinguish between very similar instances. Given a randomly selected instance *R_i_*, it searches k-nearest neighbors from the same class, and calls them “nearest hits” H, and also K-nearest neighbors from the different class, and called them “nearest miss” M. It then updates the weight *W_v_* for all attributes depending on *R_i_* (40). The process is repeated for m times and result is averaged over m iterations, the function for iteration *v* is:

$$W_{v}=W_{v}-\frac{1}{m}\text{ con }\left(A_{v},R_{i},H\right)+\frac{1}{m}\sum_{t=1,\;t\neq R_{i}}^{c}\;\frac{p\left(\tau_{t}\right)\text{ con }\left(A_{v},\;R_{i},\;M\left(C\right)\right)}{1-p\left(R_{i},\tau \right)}$$

where con (*A_v_*,*R_i_*,*H*) and con (*A_v_*, *R_i_*, *M*) are the contribution functions of nearest neighbors (hits and misses). For example, a simple contribution function can be averaging differences of feature’s value for K-Neighbors: con(*A*_*v*_,*R*_*i*_, *H*) = $\frac{1}{k}\sum_{j=1}^{k}$ diff(*A*_*v*_, *R*_*i*_, *H*_*j*_). ReliefF can efficiently evaluate features when there are strong dependencies among them (40), but like other feature filtering methods, it could not discriminate between redundant features.

For each feature ranking method, we varied the selection size from 5 to 45 (5, 10, 15, …, 45) and fit three classifier on the selected feature subset using training dataset. We then evaluate those classifiers by evaluating their prediction performance on validation cohort. Performance of each classifier is measured by AUC. All feature ranking part is performed with R package “CORElearn” in R 3.2.0 (42).

### Classifier Models

Three classifiers: random Forests, Naive Bayesian, and K-nearest neighbors were evaluated in this study.

Random Forest is an ensemble learning method for classification, which consists of a collection of decision trees (48). It uses weighted average of those trees for the final decision. This classifier is robust to noises and outliers, and can handle high dimensional spaces fast, but it has been observed to have overfitting problem (48). In this experiment, we set the number of decision trees to 100 and the number of nearest instances for weighted classification to 30.

Naive Bayes is another efficient learning algorithms for machine learning. It is a probabilistic classifier based on Bayes’s rule and strong conditional independence assumption among features. The probability of an example *E* = (*x*_1_, *x*_2_, …, *x_n_*) belonging to class *c* is defined as $p\left(c|E\right)\;=\;\frac{p\left(E|c\right)p\left(c\right)}{p\left(E\right)}$. E is classified to a positive class if and only if $f_{b}\left(E\right)\;=\;\frac{p\left(C=+|E\right)}{p\left(C=-|E\right)}\;\geq \;1$. Assume all features are independent and given the class $p\left(E|c\right)\;=\;p\left(x_{1},x_{2},\ldots x_{n}|c\right)\;=\;\prod_{i=1}^{n}\;\frac{p\left(x_{i}|C=+\right)}{p\left(x_{i}|C=-\right)}$, the Baye’s classifier is: $f_{\mathrm{nb}}\left(E\right)=\frac{p\left(C=+\right)}{p\left(C=-\right)}\prod_{i=1}^{n}\;\frac{p\left(x_{i}|C=+\right)}{p\left(x_{i}|C=-\right)}$.

Naive Bayes has advantage of requiring small amount of training data to estimate each parameter. Although Naive Bayes has strong independency assumptions, which is hardly to meet in practice, it has been shown to be effective even in cases of completely deterministic dependency among features (49).

K-nearest neighbors is another non-parametric method used for classification. It is one of the simplest learning methods. The advantage of K-nearest neighbors is that it makes no assumption about the characteristic of the features. One major problem is that it is easily misled by irrelevant features to the classification (noise) and highly susceptible to curse of dimensionality. Therefore feature selection is important before fitting this model (50). Moreover, it is computationally intensive classification method. We used *k* = 9 for the implementation of this method.

## Results

A total of 440 radiomic features were investigated in terms of their association with and power to predict tumor histology. Feature selection and classification training was done using the training cohort Lung1 (*n* = 198), and the performance was evaluated in the independent validation cohort Lung2 (*n* = 152).

**Figure 1:**
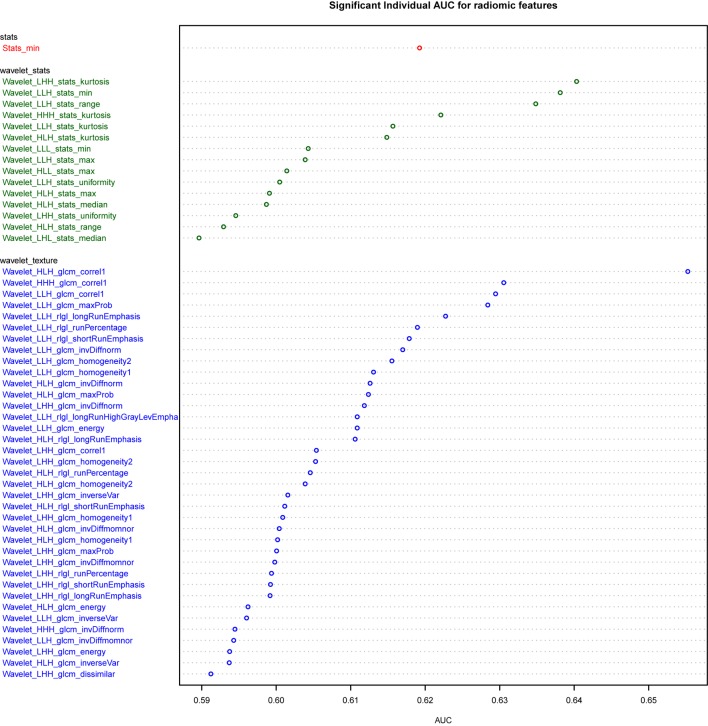
**Plot of univariate AUC for 53 significant radiomic features**.

Univariate analysis of the training dataset showing 53 features have significant predictive power (5% FDR corrected), nearly all of which are wavelet transformed features (Figure 1). Wavelet_HLH_glcm_correl1 had the highest AUC of 0.66 (CI: 0.57–0.74, *p* value 0.003). The adenocarcinoma subgroup has a higher value than the squamous carcinoma subgroup for nine gray-level cooccurrence matrix (GLCM)-based texture features (HLH and LLH wavelet transformed Energy, Homogeneity1, Homogeneity2, Inverse Variance, LLH wavelet transformed Maximum Probability), and two Gray-Level Run-Length texture features (LLH wavelet transformed Long-Run Emphasis and Long-Run High-Gray Level Emphasis). On the other hand, the squamous carcinoma subgroup has a higher value for four RLGL features (HLH and LLH wavelet-transformed Run Percentage and Short-Run Emphasis) and one statistic feature (LLH wavelet transformed Kurtosis).

**Figure 2:**
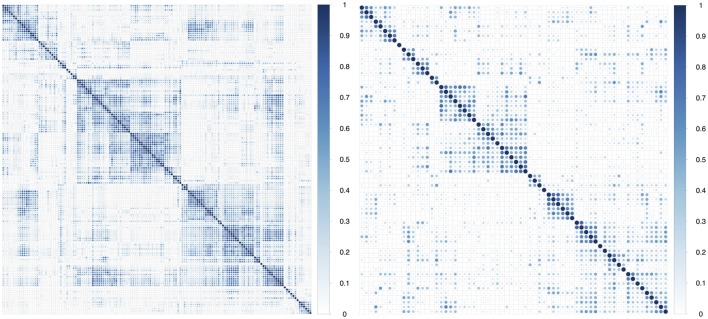
**Plot of absolute value of pair-wise Pearson correlations of radiomic features in the training dataset before and after correlation filtering**.

In multivariate analysis, we observed that about 75% of the features had absolute pair-wise Pearson correlations higher than 0.8, and 67% were over 0.9 (Table 2). To reduce redundancy, we removed features having high absolute pair-wise correlation (*C* = 0.8). The feature number was reduced from 440 to 67 after eliminating redundant features (Figure 2). The mean of the absolute pair-wise correlations was reduced from 0.36 to 0.18 and the interquartile range (IQR) for the correlations was reduced from 0.39 to 0.20.

**Table 2:** **Classification accuracy of the optimal classifier**.

Optimal cut-off	Sensitivity	Specificity	PPV	NPV	Accuracy
0.271	0.55	0.8	0.72	0.65	0.70

**Figure 3:**
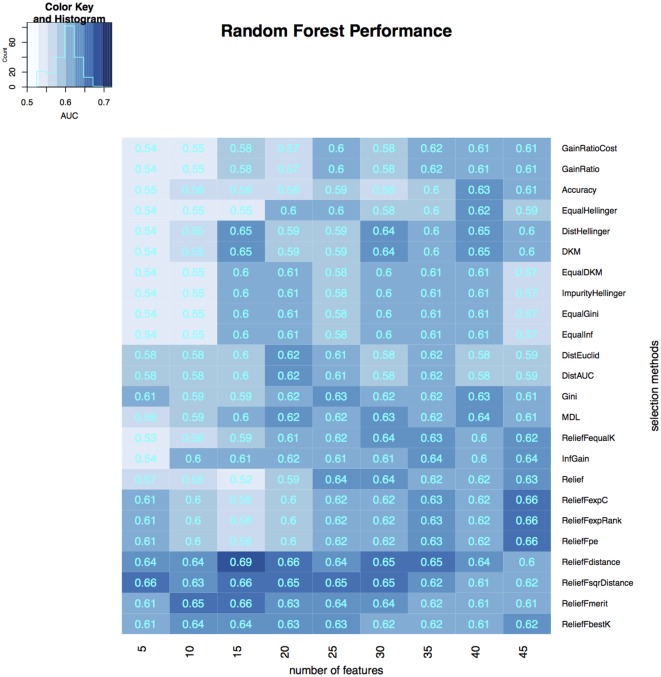
**Heatmap describing the predicative performance (AUC) of random forest in NSCLC histology classification across feature selection methods (in columns) and range of selection sizes (in rows)**.

**Figure 4:**
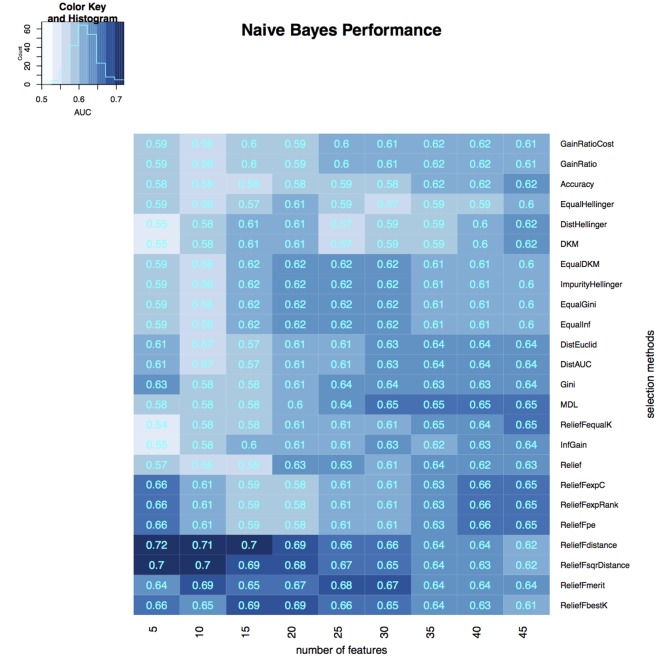
**Heatmap describing the predicative performance (AUC) of Naive Bayes in NSCLC histology classification across feature selection methods (in columns) and range of selection sizes (in rows)**.

**Figure 5:**
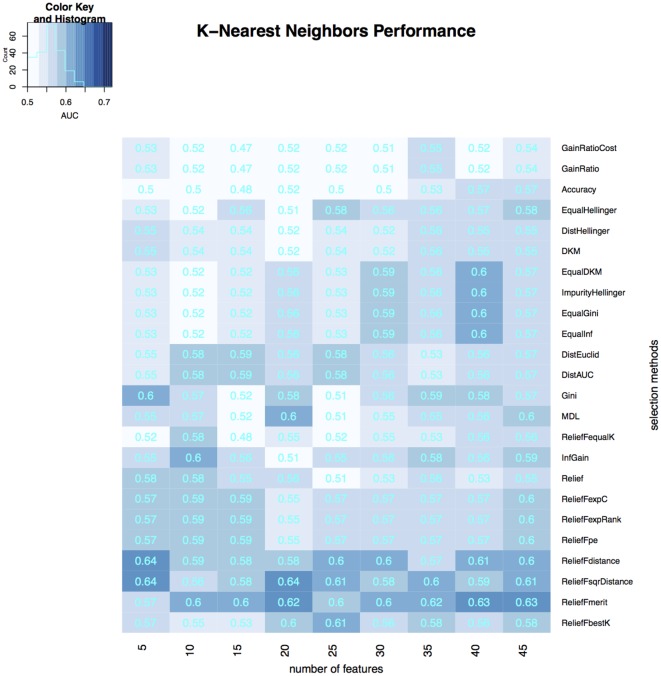
**Heatmap describing the predicative performance (AUC) of K-nearest neighbors in NSCLC histology classification across feature selection methods (in columns) and range of selection sizes (in rows)**.

To select the most relevant features, we applied 24 filtering methods to the reduced feature sets. For each filtering method, we incrementally selected 5–45 features with an increment of 5 features (5, 10, 15, …, 45). Three classifiers were then developed on selected features in the training dataset, and the classification accuracy of each classifier was tested on the validation dataset (Figures 3–5). The model with the best performance (AUC = 0.72, *p* value = 2.3 × 10^−7^) was Naive Bayes, with five predictors selected by ReliefFdistance. We obtained the optimal cutoff on the ROC curve of training cohort and used that cutoff of probability score on validation cohort to measure other prediction measures (Table 2).

As far as feature selection method is concerned, ReliefFdistance showed the highest predictive performance with all three classifiers: random Forest (AUC = 0.69), Naive Bayes (AUC = 0.72), and K-nearest neighbors (AUC = 0.64). Feature selection method ImpurityHellinger for Random forest (AUC = 0.61), Gain Ratio for K-Nearest Neighbor (AUC = 0.55), and EqualHellinger (AUC = 0.62) for Naive Bayes showed lowest predictive performance. It can be observed that features evaluated using ReliefF variants had the most favorable performances for all three classifiers (see Figures 3–5).

In order to compare overall performance the classifiers, we used the median AUC across all 24 feature selection method as a representative AUC of each classifier. Naive Bayes had the highest performance while K-Nearest Neighbor showed the lowest performance. Random Forest is the least sensitive to feature selection methods as it showed very little standard deviation in AUC (Table 3).

**Table 3:** **Table describing the median value of AUC, the optimal number of features, and AUC for best/worst features selection methods**.

	AUC (median ***±*** SD)	Optimal feature number	Best/worst feature selection method (AUC)
Naive Bayes	0.64 ± 0.028	5	ReliefFdistance (0.72)/EqualHellinger (0.62)
Random forest	0.63 ± 0.012	15	ReliefFdistance (0.69)/ImpurityHellinger (0.61)
K-Nearest neighbor	0.60 ± 0.23	20	ReliefFdistance (0.64)/gain ratio (0.55)

## Discussion

Medical imaging has the capacity to assess tissues characteristics non-invasively, and therefore it is routinely used for diagnostic and treatment purposes in cancer care. An emerging field radiomics quantifies phenotypic characteristic of tumor tissues on medical images. In this study, we investigated the association of radiomic features and the NSCLC histological subtypes. In our univariate analysis, we observed 53 features having significant association with histological subtypes. Despite the difference in the class distributions between training and validation dataset, the multivariate machine learning models achieved high prediction accuracy, which suggests the effectiveness of these advanced machine-learning approaches as well as the strong association of radiomic features and NSCLC histology.

Our study showed that ReliefF and its variants were optimal among the 24 feature selection methods assessed. Particularly, RelifFbestK, ReliefFmerit, ReliefFdistance, and ReliefFsqrDistance were consistently effective for all three classifiers. One reason for this is that the ReliefF-family does not assume the independence of features, unlike many other feature selection methods. The ReliefF algorithms are able to detect context information among features and thus more accurately deals with situations where dependencies are present (51). However, like other feature selection methods, ReliefF is also unable to detect redundant features which tend to have similar scores for evaluation (52). We took care of this problem by performing correlation-based feature elimination before feature selection stage.

For the performance of three classifiers, Naive Bayes performs better than Random Forest and K-nearest neighbors. Although the median performance across feature selection methods was about the same for Naive Bayes and Random Forest, the best model achieved by Naive Bayes (AUC: 0.72; *p* value = 2.3 × 10^−7^) was higher than Random Forest (AUC: 0.68; *p* value = 1.38 × 10^−5^). K-nearest neighbors has the lowest performance among the three classifiers, and ReliefF could only slightly improve its accuracy. This may because K-nearest neighbors is very sensitive to noise (50).

A shortcoming of our study is that the cut-off *M* = 0.8 is arbitrarily chosen in the correlation elimination part. One could set a more stringent or relaxed threshold based on the degree of redundancy in the dataset. A better approach would use a range of thresholds, combine them with second stage feature selection, and choose one with the most favorable result. Additionally, this two-stage feature selection is expected to fail when the interaction of two non-informative features has strong predictive power. In this case, a multivariate feature selection method like mRMR is more suitable (35).

Recently, Parmar et al. (22) identified and validated cancer specific radiomic feature clusters using consensus clustering, which provided an important tool to enhance the feature selection process. Their study found radiomic features’ association with histology using an unsupervised method and achieved AUC = 0.64 for prediction. In our study by using supervised feature selection methods, we achieved higher AUCs. In another study, Basu et al. used decision trees and support vector machines for tumor classification, and results showed that ReliefF outperformed wrapper methods for 2D radiomic features (28). However, unlike our study, their results were based on a smaller cohort (*n* = 74) and lacked independent validation due to the unavailability of an independent validation cohort. Lastly Zhang et al. presented a two-stage feature selection method by combining ReliefF and mRMR (31). They showed that such a hybrid feature selection approach could improve the effectiveness of gene selection, and this could provide better discrimination for biological subtypes. This new algorithm’s ability in selecting radiomic features for histology classification should be evaluated in further studies.

It should also be noted that retrospective cohorts-based radiomic studies like this, face many challenges. Radiomic features are sensitive to the variability of imaging scanners and scanning parameters (53, 54), tumor delineation methods (55, 56), image reconstruction (57), discretization techniques (58), etc. These different factors are not controlled for retrospective cohorts, which maybe one of the reason for not so impressive performance. Prospective cohorts created by controlling these factors could provide higher performance. In future studies, the performance of classifiers can be enhanced if we incorporate genetic signatures and clinical features like tumor grade, location, smoking history, and obesity (59, 60). For example, a recent study showed that body mass index was inversely associated with squamous carcinoma, yet for adenocarcinoma the association was positive (60). It is also important to take genetic heterogeneity into account. Recent studies examining gene expression profiles have identified several genes distinguishing adenocarcinoma and squamous carcinoma (61–63). Hence, future study incorporating clinic characteristics and genomic data will improve classification accuracy.

In conclusion, radiomic features have strong predictive power for classification of NSCLC histology. With expanding cohorts and improving image feature extraction techniques, we expect higher accuracy in classification using radiomics. This work will serve as a promising diagnostic tool for identifying lung cancer histology in a non-invasive way and thus informing treatment choices and personalized therapy for lung cancer patients.

## Author Contributions

HA, WW, and CP conceived of the project, analyzed the data, and wrote the paper. PG, JQ, JB, PL, and RM provided expert guidance, data, or analysis tools, and reviewed the manuscript.

## Conflict of Interest Statement

The authors declare that the research was conducted in the absence of any commercial or financial relationships that could be construed as a potential conflict of interest.
